# Retrorectal Tailgut Cyst: A Case Report

**DOI:** 10.7759/cureus.23319

**Published:** 2022-03-19

**Authors:** Neel Shah, Peter Edelstein

**Affiliations:** 1 Medicine, University of Central Florida College of Medicine, Orlando, USA; 2 Colorectal Surgery, University of Central Florida College of Medicine, Orlando, USA

**Keywords:** presacral tumor, retrorectal, tailgut, kraske approach, cystic hamartoma, retrorectal tumor, tailgut cyst

## Abstract

Tailgut cysts, or retrorectal cystic hamartomas, are rare congenital abnormalities that develop when the embryologic tailgut fails to involute. They are found in the presacral space, which is an area with quite a complex anatomy. Tailgut cysts can be symptomatic due to their mass effect and can even result in complications, including malignancy. Because of their rarity and varied presentations, tailgut cysts are frequently misdiagnosed. CT scans and MRI are useful in the diagnosis of these retrorectal masses, and surgical resection is the definitive treatment. Multiple surgical approaches can be used, with the treatment tailored to suit each individual patient’s anatomy and suspected lesion diagnosis.

## Introduction

The retrorectal or presacral space is bounded anteriorly by the rectum, posteriorly by the sacrum, superiorly by the peritoneal reflection, inferiorly by the pelvic diaphragm muscles (levator ani and coccygeus), and laterally by the ureters and iliac arteries and veins [1]. Tailgut cysts or retrorectal cystic hamartomas present in the retrorectal space, an area with complex embryology and anatomy, which offers the potential for a wide variety of benign and malignant pathology [1,2]. Tailgut cysts are rare congenital malformations that are embryologic remnants of the postnatal component of the hindgut [3]. As the embryo folds during the fourth week of gestation, the cloacal membrane progresses ventrally and encloses a portion of the future gut that is distal to the eventual hindgut - a region known as the “tailgut” [3]. Typically, the tailgut involutes around the sixth week of gestation; however, when this process fails, a tailgut cyst remains [3].

Tailgut cysts are quite rare, but the true incidence has been difficult to quantify as the majority are asymptomatic and can easily be missed on the digital rectal exam [3,4]. Most patients present between the ages of 30 and 60 years, and there is a strong female predominance, likely occurring three to five times as often in women as in men [1,3,5]. When tailgut cysts do cause symptoms, these are normally due to the mass effect of the lesion on adjacent organs, cyst infection, or malignant transformation [1,2,4,5]. Mass effect can lead to such symptoms as constipation, tenesmus, dyschezia, and/or polyuria; and lesion infection may manifest as a perianal fistula or a pelvic abscess [1-3,5]. Based on recent studies, the overall rate of malignant transformation of tailgut cysts is estimated to be as high as 26% [6].

In this report, we present the case of a woman with a tailgut cyst that caused lower back pain, whose symptoms were successfully relieved with surgical resection.

## Case presentation

**Figure 1:**
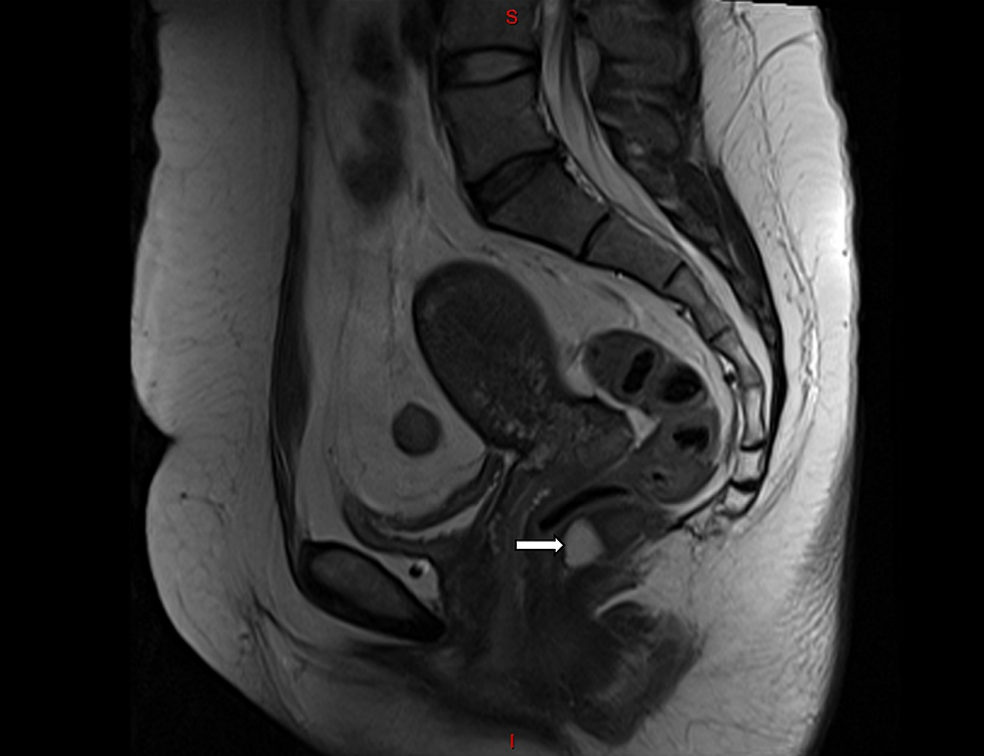
Sagittal MRI of the pelvis: cystic, multi-loculated retrorectal mass (arrow) MRI: magnetic resonance imaging

A 46-year-old woman with an uneventful past medical and surgical history was evaluated for lower back pain. Her diagnostic workup included an MRI of the lumbar spine and sacrum. This imaging study demonstrated an extremely low-lying (caudal), multi-loculated retrorectal lesion 3.7 cm in its greatest dimension, most likely representing a tailgut cyst (cystic hamartoma) (Figure 1). After being informed about the lesion on her imaging study, the patient relayed that she occasionally felt pain around her tailbone (coccyx) when sitting.

Digital rectal examination immediately demonstrated the presence of a caudal, tender, soft, non-mobile retrorectal mass. The examining finger was able to pass superiorly to the mass and palpate a normal mid-S5 sacral vertebra. Perianal examination revealed a normal resting tone, normal squeeze, and no perineal descent or prolapse with Valsalva; furthermore, there was no gross perianal pathology. Aside from these findings, the remainder of the physical exam was unremarkable. Laboratory studies were within normal limits, and there was no radiographic evidence of extra-pelvic disease.

The patient underwent complete resection of the mass through a posterior (Kraske) approach. An incision was made extending 6 cm from the coccyx superiorly. Sharp dissection was used to carry the incision down directly in the midline until the presacral fascia was encountered. The medial gluteal fibers were then divided bilaterally to expose the distal sacrum and attached mass. The mass had multiple tense and cystic loculations, one of which was inadvertently entered, releasing a thick, non-malodorous, purulent fluid consistent with sterile pus. The lesion was resected en bloc with its fibrous attachments to the distal coccyx and inferior S5 sacral vertebra by dividing across the mid S5 sacral vertebra. After the removal of the mass, orthopedic files and a rongeur were used to dull the sharp edge of the sacral transection, and bone wax was placed to prevent additional bleeding or soft tissue injury. The gluteal muscles were returned to the midline, and a Jackson-Pratt drain was cut to size and passed into the presacral space through a separate, lateral skin site. The remaining layers of the incision were re-approximated and closed. The patient tolerated the operation without any complications, and her postoperative course was uneventful. The patient returned to the clinic two weeks later for the removal of the drain and skin sutures and achieved a complete functional recovery soon thereafter.

Pathological findings

**Figure 2:**
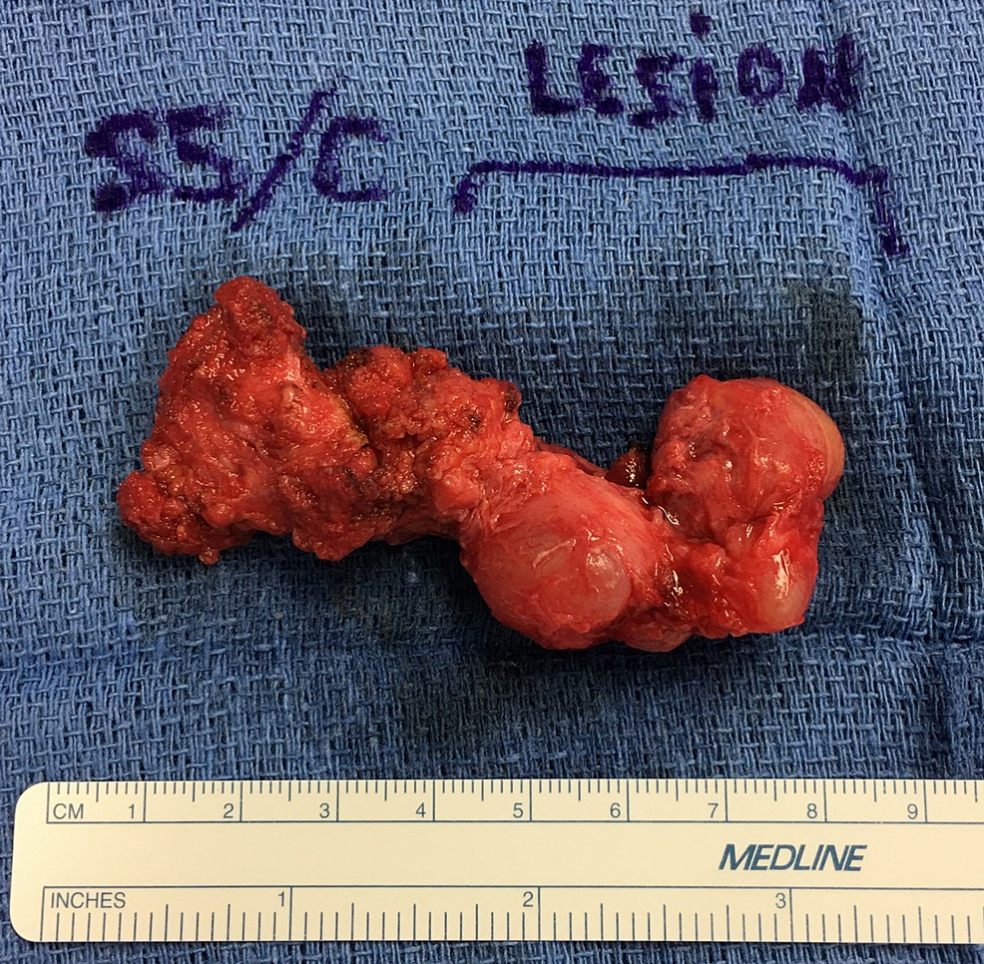
Gross image of en bloc resection including irregularly shaped cysts

Gross pathological examination revealed an irregular, rubbery, firm, tan-red mass of tissue measuring 5.5 x 3.7 x 2.6 cm with attached coccyx and inferior S5 sacral vertebra (Figure 2). The mass appeared to be comprised of several cysts ranging in size from 0.3 to 2 cm in maximum dimension, all of them containing jelly-like contents. The lining of the cysts was smooth and glistening.

Microscopically, the mass demonstrated columnar and squamous epithelium with no cellular atypia or significant mitotic activity. The cyst wall consisted of fibromuscular tissue with no desmoplastic changes. There was no evidence of malignancy, and the lesion was confirmed to be a benign retrorectal tailgut cyst.

## Discussion

Tailgut cysts are frequently misdiagnosed due to the frequent absence or vagary of symptoms and their rarity; therefore, the first diagnostic move is simply to include the possibility of such a lesion on the differential diagnosis [2]. Tailgut cysts have a wide variety of presentations and can often mimic symptoms more commonly associated with other conditions; for example, difficulty with defecation or urinary retention can result from large cysts [1,3-5]. Unusual symptomatic presentations can also occur, such as sciatica [3]. Complications from mass effect and infection can be the primary indication of a tailgut cyst [1,3]. Finally, malignant transformation into neuroendocrine tumors, adenocarcinoma, and squamous cell carcinoma can occur; thus, accurate diagnosis and treatment are critical [1-3,7].

Imaging, namely MRI and CT scans, is the principal method by which retrorectal masses can be diagnosed [8]. Other imaging modalities can be useful but are not diagnostic [8]. On MRI, tailgut cysts may demonstrate hypointensity on T1-weighted images and hyperintensity on T2-weighted images (although, this is not always the case, as cyst contents can vary) [8]. Fine-needle aspiration has been demonstrated to be successful but is not considered standard due to the potential risks, and current consensus calls for surgical resection of the diagnosed mass with subsequent gross pathological and histopathological evaluation [8].

Surgical resection is recommended for both asymptomatic lesions (due to risks including malignant transformation) and symptomatic tailgut cysts [3,5,7]. Approaches may vary widely, from laparoscopic to transabdominal to transsacral to transanal, and each approach offers a unique set of potential benefits and drawbacks [3]. Low-lying (caudal) retrorectal masses (entirely below S3) can safely be accessed via a posterior, or Kraske, approach, avoiding entry into the peritoneal cavity; however, this approach is generally not recommended for more cranially located lesions [3]. These higher lesions (entirely above S3) can be excised via an anterior, or abdominal, approach [9]. When lesions lie both above and below the level of S3, an integrated abdominosacral approach may be warranted [9]. When the posterior approach is employed, a coccygectomy may be performed for better surgical exposure [3]. If the tailgut cyst is not resected entirely, there is a possibility for recurrence [3]. Many treatment options have been explored, but en bloc resection with clear margins is the most effective one and is associated with decreased incidence of recurrence and improved patient outcomes [3,10]. Following excision, an annual digital rectal exam is recommended; furthermore, a CT scan is recommended in postoperative years one and five [3].

## Conclusions

Despite the rarity of tailgut cysts, it is important for clinicians to be alert to recognize these lesions’ clinical features. Because symptoms can range widely, physicians must keep tailgut cysts on their differential diagnosis; additionally, physicians must have a high degree of suspicion because of the cyst's potential for malignant transformation. Being aware of and recognizing these lesions can help in applying rapid surgical intervention for definitive treatment, as well as decreased patient morbidity and mortality.
